# Prognostic clinical factors in pretreated colorectal cancer patients receiving regorafenib: Implications for clinical management

**DOI:** 10.18632/oncotarget.5053

**Published:** 2015-08-17

**Authors:** Michela Del Prete, Riccardo Giampieri, Fotios Loupakis, Tiziana Prochilo, Lisa Salvatore, Luca Faloppi, Maristella Bianconi, Alessandro Bittoni, Giuseppe Aprile, Alberto Zaniboni, Alfredo Falcone, Mario Scartozzi, Stefano Cascinu

**Affiliations:** ^1^ Medical Oncology, AO Ospedali Riuniti-Università Politecnica delle Marche, 60126 Ancona, Italy; ^2^ Medical Oncology, Fondazione Poliambulanza, 25124 Brescia, Italy; ^3^ Medical Oncology, Azienda Ospedaliero-Universitaria Pisana, 56126 Pisa, Italy; ^4^ Medical Oncology, Azienda Ospedaliero-Universitaria “S. Maria della Misericordia”, 33100 Udine, Italy; ^5^ Medical Oncology, Azienda Ospedaliero-Universitaria di Cagliari, 09042 Monserrato (CA), Cagliari, Italy

**Keywords:** regorafenib, colorectal cancer, prognostic factors, LDH, neutrophil/lymphocyte ratio

## Abstract

**Background:**

We assessed the impact on survival of angiogenesis and inflammation-related factors, particularly LDH serum levels, platelet, neutrophil and lymphocyte counts, and neutrophil-to-lymphocyte ratio (NLR), in metastatic colorectal cancer patients receiving regorafenib monotherapy.

**Methods:**

LDH serum levels, neutrophil, lymphocyte and platelet counts were collected at the start of regorafenib monotherapy. Cut-off values were calculated by ROC curve analysis. Survival analyses were performed by Kaplan-Meier method, and multivariate analysis by Cox method.

**Results:**

A total of 208 patients were eligible for analysis. Among factors who were related with worse overall survival and who maintained their role at the multivariate analysis, high platelet count (Exp(b):1.4963, 95% CI:1.0130–2.2103, *p* = 0.0439) and high neutrophil/lymphocyte ratio (Exp(b):1.6963, 95% CI:1.0757–2.6751, *p* = 0.0237) were those who more deeply were related to worse overall survival. High lymphocyte count (Exp(b):0.4527, 95% CI:0.2801–0.7316, *p* = 0.0013) was correlated with improved overall survival.

**Conclusions:**

High neutrophil, high platelet, low lymphocyte count and/or high NLR may represent negative prognostic factors in patients receiving regorafenib monotherapy. It is advisable that these factors are taken into account in the design of subsequent trials in colorectal cancer patients receiving this drug.

## INTRODUCTION

The introduction of regorafenib represented a significant step forward in the treatment of metastatic colorectal cancer (mCRC) patients. This novel oral multikinase inhibitor improved both progression free survival (PFS) and overall survival (OS) in heavily pre-treated patients otherwise not suitable for any further active therapy [1]. However, the absence of predictive factors for treatment efficacy/resistance along with the distinct toxicity profile of this novel compound, requires a very well balanced clinical assessment of the risk to benefit ratio in each patient potentially candidate to such a treatment approach. Indeed, even in carefully selected cases, available data seem to indicate that a not negligible proportion of patients does not derive any benefit from this treatment and is thus exposed to unnecessary toxicity.

It is hypothised that tumour-driven neoangiogenesis represents the main biological target of regorafenib: molecular mechanisms underlying angiogenesis may be then considered as important predictive factors for clinical outcome during regorafenib treatment.

The biological link between hypoxia, lactic dehydrogenase (LDH) levels and the tumour-driven angiogenesis pathway through the abnormal activation of the Hypoxia Inducible Factor 1 α (HIF1-α) is well established [2, 3]. Since LDH and pro-angiogenesis factors are regulated by the same HIF1α-driven molecular pathway, high LDH levels are concomitantly present along with abnormal activation of the VEGF pathway [4]. Accordingly to this biological assumption, Azuma et al [5] demonstrated that high LDH serum levels were associated with tumour over-expression of VEGFA and VEGFR-1. As a clinical consequence it has been speculated that LDH levels may represent an indirect indicator of activated tumour angiogenesis and worse prognosis [6–7]. In CRC, a significantly correlation has been demonstrated between LDH overexpression and increased risk of metastases, and between high LDH serum levels and worse prognosis [8–9]. The role of LDH in CRC patients receiving anti-angiogenic therapy is more controversial. In an exploratory analysis of the CONFIRM-1 and 2 trials, median PFS resulted improved with the use of PTK/ZK (vatalanib, an oral inhibitor of VEGF-receptors) in the subgroup of patients with high serum LDH [10–11]. Recently Koukourakis et al also demonstrated that serum LDH and tissue LDH-5 are complementary features that may help characterizing the activity of LDH in CRC [12]. Accordingly with these observations we previously suggested that pre-treatment LDH levels may represent a relevant factor for the prediction of bevacizumab efficacy in an unselected population of CRC patients [13].

Neutrophil, lymphocyte and platelet count have been identified as markers of clinical outcome in different tumour types, in particular in heavily treated mCRC patients.

Leucocytes, including neutrophils and lymphocytes, were reported to play an important role in tumour inflammation and immunology [16] and to have a prognostic value in patients with cancer [14–17]. In particular, the neutrophil-to-lymphocyte ratio (NLR) is a combined indicator of inflammation and immunology activity. In recent years, several studies evaluated the prognostic role of NLR in many tumour types, including CRC, suggesting that an elevated NLR could be related to an adverse outcome [17–23]. The results from a recently published meta-analysis indicated that a high pre-treatment NLR was significantly related to poorer OS and PFS [23]. Recent studies also evaluated the correlation between inflammation and tumour response to chemotherapy in various tumour types [24–26]. Lissoni et al suggested that a decreasing lymphocyte count during chemotherapy might be correlated with tumour progression. On the contrary lymphocyte count at the end of chemotherapy was significantly higher than before treatment in patients achieving an objective tumour response [27]. An elevated NLR has been shown to represent a predictive factor for clinical outcome in patients with liver-only colorectal metastases receiving neo-adjuvant chemotherapy [28]. Accordingly Chua et al demonstrated an independent predictive value in terms of clinical benefit, PFS and OS for pre-treatment NLR in patients with unresectable mCRC undergoing first-line chemotherapy [29]. Many clinical and pre-clinical findings also underscored the role of platelets in different tumour related events. Activated platelets, in response to tissue impairment, can induce a pro-inflammatory response leading to increased concentrations of pro-inflammatory and pro-tumorigenic factors [30]. Globally the potential link between systemic inflammation and tumour angiogenesis and thus the hypothetical predictive role of inflammatory markers during antiangiogenetic therapies has been evaluated in CRC patients treated with bevacizumab with interesting insights into the biological mechanisms underlying the interaction between inflammation, angiogenesis and anti-VEGF therapy [31].

Aim of the present study was to assess the role of angiogenesis and inflammation-related factors such as LDH serum levels, platelet count, neutrophil and lymphocyte count, and NLR in predicting clinical outcome for pre-treated mCRC patients receiving regorafenib. This was done in order to individuate potentially reliable and easy to use markers for patients stratification and selection.

## RESULTS

### Univariate analysis

Globally 208 patients were available for our analysis. Median age at the start of treatment was 61 (Range 32–90). All major clinical characteristics have been summarised in table 1. In the global population median OS was 3.5 months whereas median PFS was 2.4 months. Among 202 patients who were assessable for response, 10 (5%) achieved at least a partial response, 58 (29%) obtained disease stabilization and the remaining 134 (66%) showed progressive disease.

**Table 1:** Main clinical characteristics of the patients population, according to pre-treatment LDH values, platelets count, neutrophil and lymphocyte count, and neutrophil/lymphocyte ratio (≥or < than the cut-off levels)

			LDH serum level *(cutoff 1.21 ULN)*			Neutrophil count *(cutoff 0.96 ULN)*			Lymphocyte count *(cutoff 1.77 LLN)*			Platelet count *(cutoff 0.54 ULN)*			Neutrophil to Lymphocyte Ratio *(cutoff 0.38)*		
		*n* = 208	*≥ cutoff* (*n* = 95)	*< cutoff* (*n* = 113)	*p value*	*≥ cutoff* (*n* = 50)	*< cutoff* (*n* = 158)	*p value*	*≥ cutoff* (*n* = 64)	*< cutoff* (*n* = 144)	*p value*	*≥ cutoff* (*n* = 92)	*< cutoff* (*n* = 116)	*p value*	*≥ cutoff* (*n* = 126)	*< cutoff* (*n* = 82)	*p value*
Age (range)		61 (32–90)	56 (32–80)	65 (40–90)	ns	59 (38–80)	61 (32–90)	ns	63 (36–90)	59 (32–86)	ns	62.5 (35–90)	55 (32–78)	ns	59.5 (32–87)	63.5 (37–90)	ns
**Sex**	M F	103(49%) 105(51%)	41(43%) 54(57%)	62(55%) 51(45%)	ns ns	21(42%) 29(58%)	82(52%) 76(48%)	ns ns	29(45%) 35(55%)	74(51%) 70(49%)	ns ns	43(47%) 49(53%)	60(52%) 56(48%)	ns ns	61(48%) 65(52%)	42(51%) 40(49%)	ns ns
**K-ras mutation**	no yes	135(65%) 73(35%)	60(63%) 35(37%)	75(66%) 38(34%)	ns ns	33(66%) 17(34%)	102(65%) 56(35%)	ns ns	41(64%) 23(36%)	94(65%) 50(35%)	ns ns	55(60%) 37(40%)	80(69%) 36(31%)	ns ns	86(68%) 40(32%)	49(60%) 33(40%)	ns ns
**Adjuvant chemotherapy**	yes no	89(43%) 119(57%)	43(45%) 52(55%)	46(41%) 67(59%)	ns ns	24(47%) 26(53%)	65(41%) 93(59%)	ns ns	26(41%) 38(59%)	63(44%) 81(56%)	ns ns	40(44%) 52(56%)	49(42%) 67(58%)	ns ns	50(40%) 76(60%)	39(48%) 43(52%)	ns ns
**Metastatic sites**	1 ≥ 2	44(21%) 164(79%)	27(28%) 68(72%)	17(15%) 96(85%)	ns ns	13(25%) 37(75%)	31(20%) 127(80%)	ns ns	13(20%) 51(80%)	31(22%) 113(78%)	ns ns	21(23%) 71(77%)	23(20%) 93(80%)	ns ns	34(27%) 92(73%)	10(12%) 72(88%)	ns ns
**Number of previous systemic anticancer therapies**	1–2 3 ≥ 4	23(11%) 52(25%) 133(64%)	15(16%) 27(28%) 53(56%)	8(7%) 25(22%) 80(71%)	ns ns ns	5(10%) 13(26%) 32(64%)	18(11%) 39(25%) 101(64%)	ns ns ns	8(13%) 13(21%) 43(66%)	15(10%) 39(27%) 90(63%)	ns ns ns	14(15%) 18(20%) 60(65%)	9(8%) 34(29%) 73(63%)	ns ns ns	11(9%) 30(24%) 85(67%)	12(15%) 22(27%) 48(58%)	ns ns ns
**Previous anti-VEGF treatment with bevacizumab**	Yes No	191(92%) 17(8%)	91(96%) 4(4%)	100(88%) 13(12%)	ns ns	45(90%) 5(10%)	146(92%) 12(8%)	ns ns	59(93%) 5(7%)	132(92%) 12(8%)	ns ns	81(88%) 11(12%)	110(95%) 6(5%)	ns ns	118(94%) 8(6%)	73(89%) 9(11%)	ns ns
**Response Rate**	PR SD PD	10(5%) 58(28%) 140(67%)	2(2%) 36(38%) 57(60%)	8(7%) 22(19%) 83(74%)	ns ns ns	3(6%) 13(25%) 34(69%)	7(4%) 45(28%) 106(68%)	ns ns ns	3(4%) 17(26%) 44(70%)	7(5%) 41(29%) 96(66%)	ns ns ns	8(8%) 31(34%) 53(58%)	2(2%) 27(23%) 87(75%)	ns ns ns	5(4%) 42(33%) 79(63%)	5(6%) 16(20%) 61(74%)	ns ns ns
**Survival (months)**	PFS OS	2.4 3.5	1.7 3.3	2.5 7.6	< 0.0001 < 0.0001	1.5 2.9	2.5 5.2	< 0.0001 < 0.0001	2.7 11.08	2.3 3.3	0.0005 < 0.0001	2.0 3.2	2.6 6.2	0.0001 < 0.0001	2.1 3.1	3.4 9.8	< 0.0001 < 0.0001

The cut-off point with the highest sensitivity and specificity for estimating pre-treatment LDH serum levels, neutrophil level, lymphocyte level, platelet count and NLR as a function of treatment clinical activity was set at 1.21 ULN, 0.96 ULN, 1.77 LLN, 0.54 ULN and 0.38 respectively after ROC analyses.

**Figure 1:**
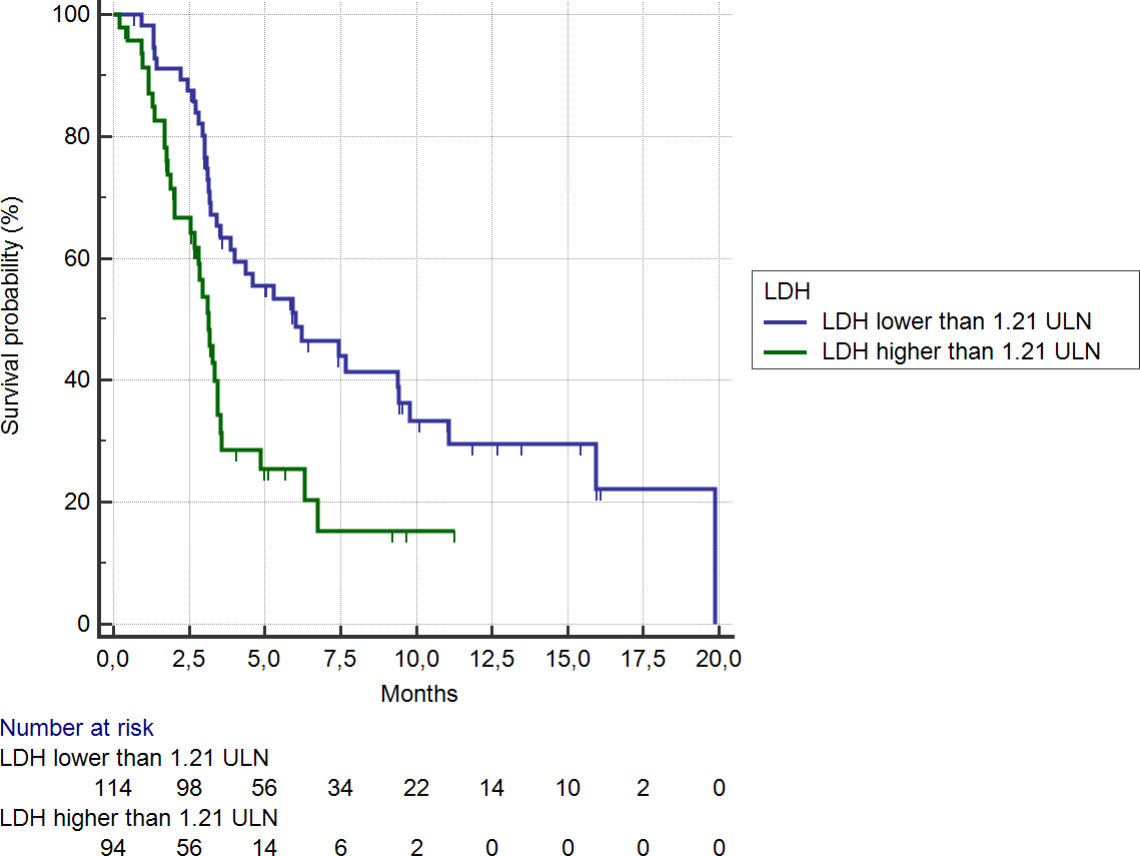
Kaplan-Meier curves for median overall survival (OS) in pre-treated metastatic colorectal cancer patients receiving regorafenib according to LDH pre-treatment level < (**———**) or ≥ (**———**) than 1.21 ULN (the cut-off value determined by ROC curve analysis) (7.6 months vs. 3.3 months; HR = 0.43, 95%CI: 0.25–0.53, *p* < 0.0001)

**Figure 2:**
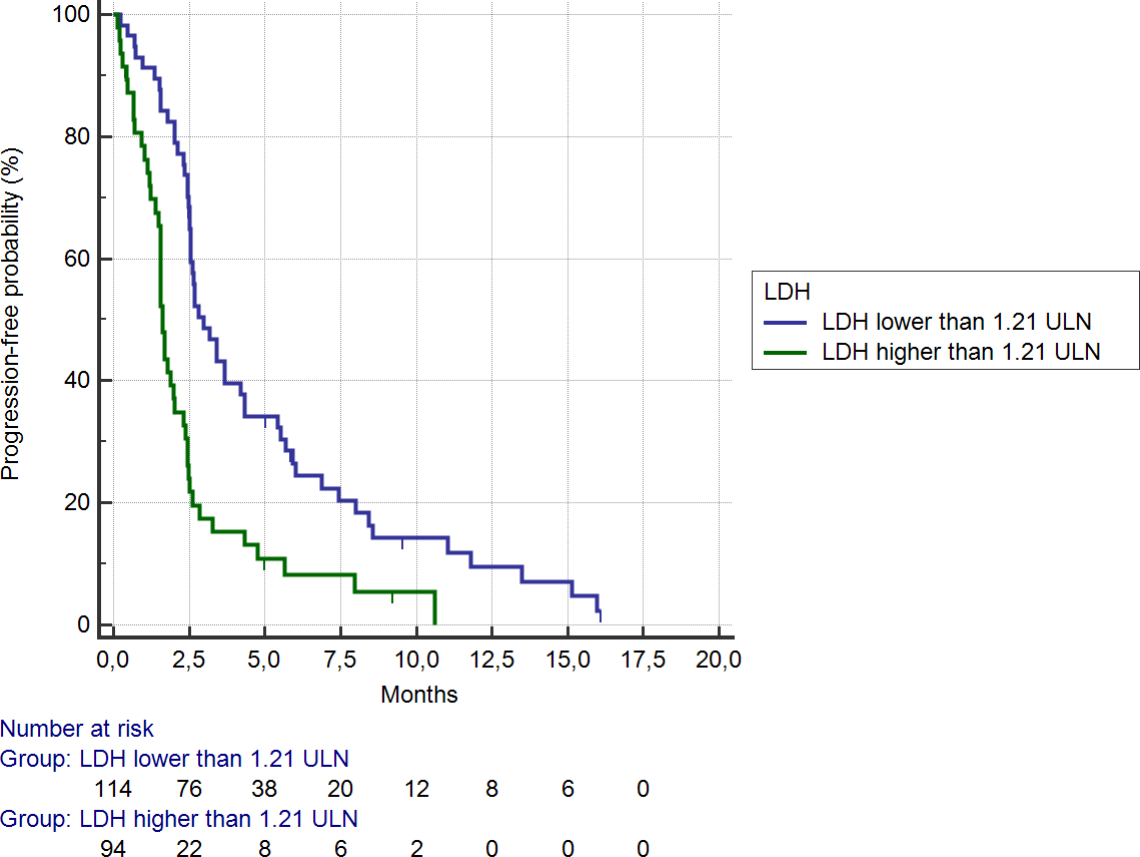
Kaplan-Meier curves for median progression free survival (PFS) in pre-treated metastatic colorectal cancer patients receiving regorafenib according to LDH pre-treatment serum level < (**———**) or ≥ (**———**) than 1.21 ULN (the cut-off value determined by ROC curve analysis) (2.5 months vs. 1.7 months; HR = 0.48, 95%CI: 0.30–0.59, *p* < 0.0001)

Among the 95 (46%) patients showing LDH serum levels ≥ 1.21 ULN, median OS was significantly shorter than among the remaining 113 (54%) patients (3.3 months vs. 7.6 months, HR: 0.43, 95% CI: 0.25–0.53, *p* < 0.0001) (Figure 1). Accordingly, a statistically significant difference was evident for median PFS (1.7 months vs. 2.5 months respectively in the LDH levels ≥ vs < 1.21 ULN group; HR: 0.48, 95% CI: 0.30–0.59, *p* < 0.0001) (Figure 2).

**Figure 3:**
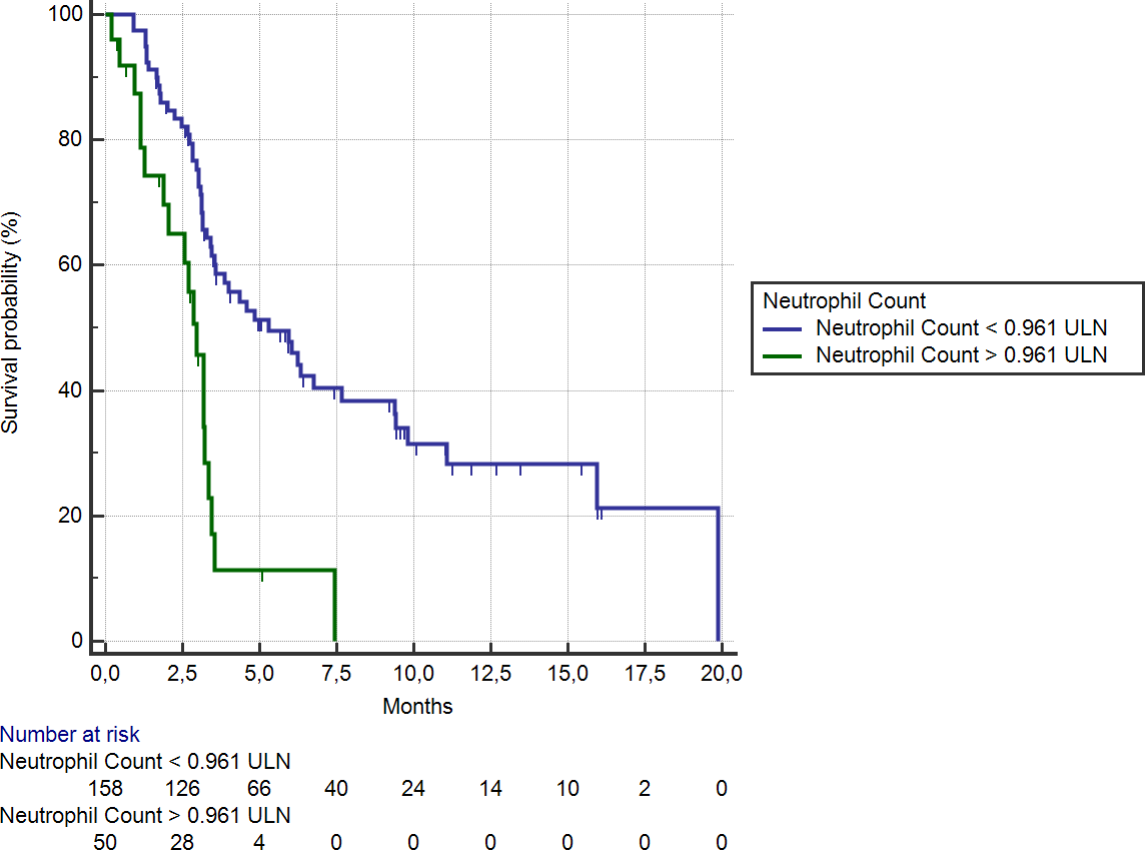
Kaplan-Meier curves for median overall survival (OS) in pre-treated metastatic colorectal cancer patients receiving regorafenib according to pre-treatment neutrophil count < (**———**) or ≥ (**———**) than 0.96 ULN (the cut-off value determined by ROC curve analysis) (5.2 months vs. 2.9 months; HR = 0.35, 95%CI: 0.12–0.35, *p* < 0.0001)

Fifty patients (24%) showed a neutrophil level ≥ 0.96 ULN. In these patients median OS was significantly shorter than among the remaining 158 (76%) patients (2.9 months vs. 5.2 months respectively; HR:0.35, 95% CI: 0.12–0.35, *p* < 0.0001) (Figure 3). Accordingly a statistically significant difference was evident for median PFS in patients showing neutrophil level ≥ or < than 0.96 ULN (1.5 months vs. 2.5 months respectively; HR: 0.42, 95% CI: 0.18–0.44, *p* < 0.0001).

**Figure 4:**
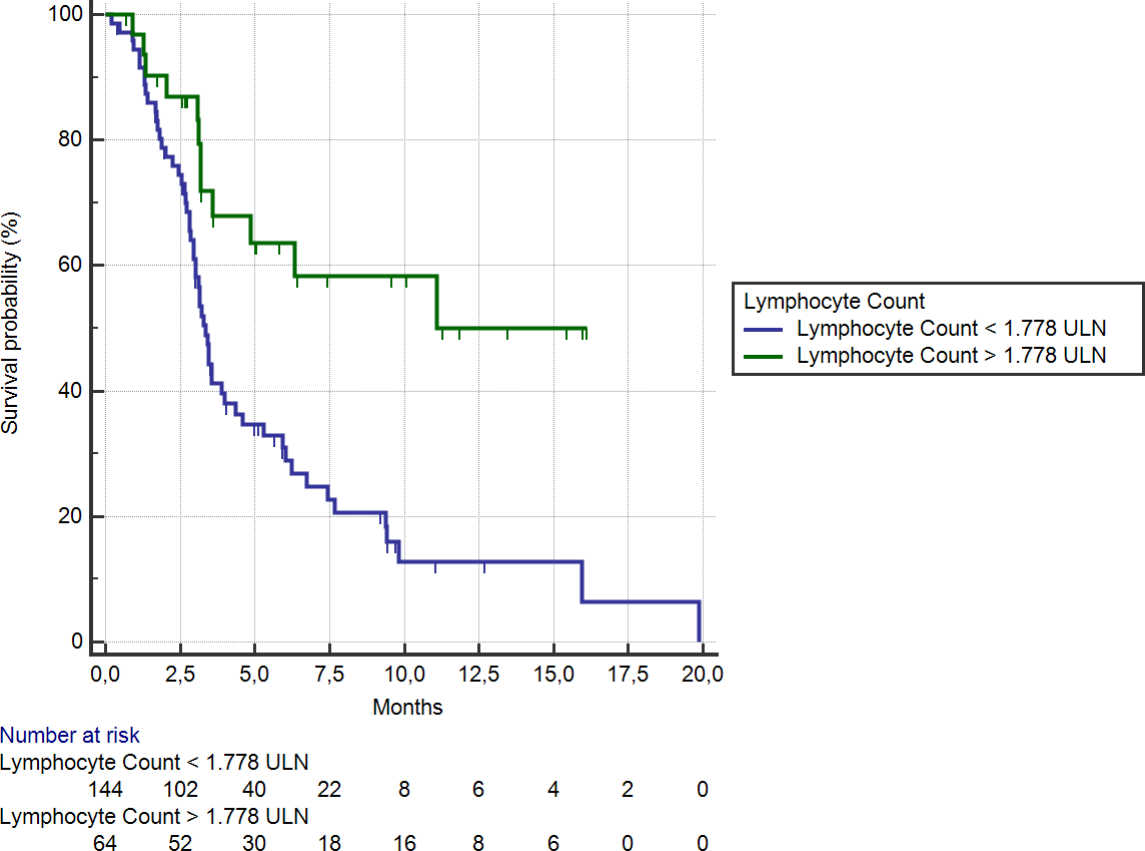
Kaplan-Meier curves for median overall survival (OS) in pre-treated metastatic colorectal cancer patients receiving regorafenib according to pre-treatment lymphocyte count < (**———**) or ≥ (**———**) than 1.77 LLN (the cut-off value determined by ROC curve analysis) (3.3 months vs. 11.08 months; HR = 2.73, 95% CI: 1.67–3.41, *p* < 0.0001)

Among the 64 (31%) patients showing lymphocyte level ≥ 1.77 LLN, median OS was significantly better than among the remaining 144 (69%) patients (11.08 months vs. 3.3 months respectively; HR: 2.73, 95%CI: 1.67–3.41, *p* < 0.0001) (Figure 4). A statistically significant difference was also evident for median PFS in patients showing lymphocyte level ≥ or < than 1.77 LLN (2.7 months vs. 2.3 months respectively; HR:1.66, 95% CI: 1.26–2.33, *p* = 0.0005).

**Figure 5:**
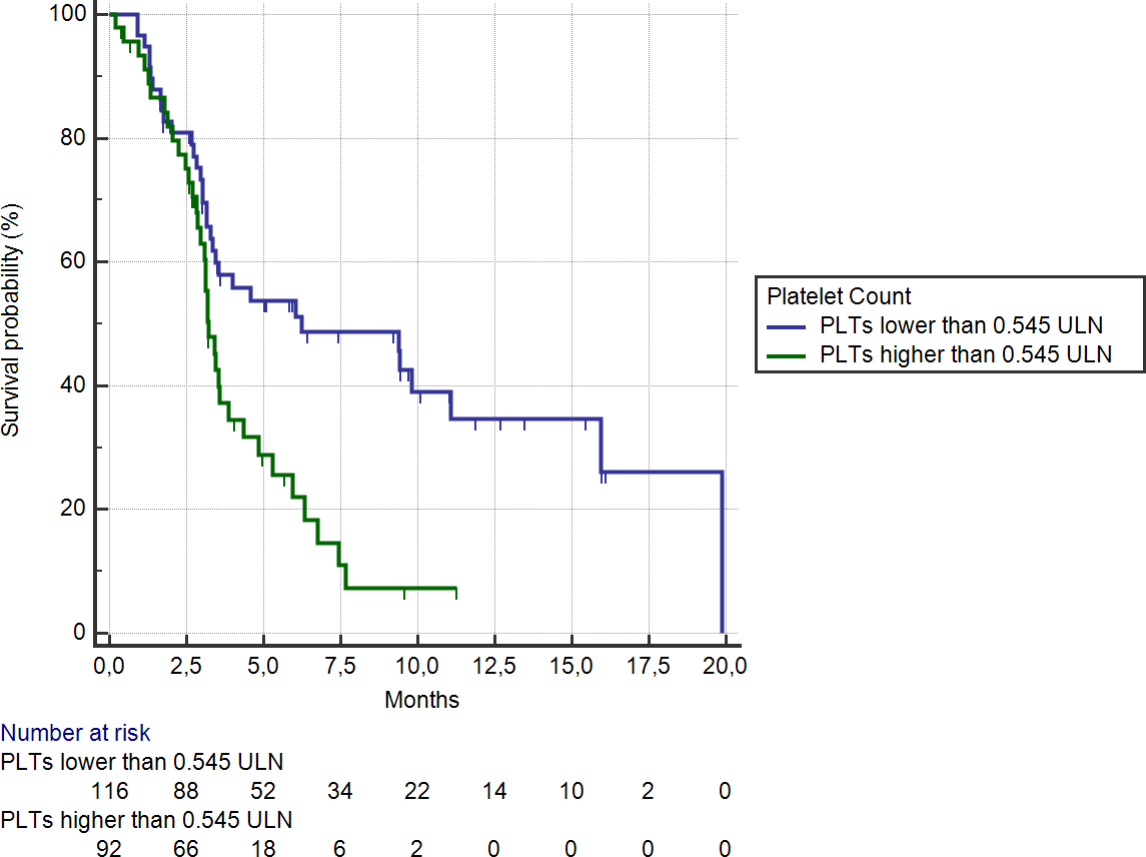
Kaplan-Meier curves for median overall survival (OS) in pre-treated metastatic colorectal cancer patients receiving regorafenib according to pre-treatment platelets count < (**———**) or ≥ (**———**) than 0.54 ULN (the cut-off value determined by ROC curve analysis) (6.2 months vs. 3.2 months; HR = 0.50, 95% CI: 0.31–0.65, *p* < 0.0001)

Among 92 (44%) patients showing a platelet level ≥ 0.54 ULN, median OS was 3.2 months, whereas in the remaining 116 (56%) patients median OS was 6.2 months (HR: 0.50, 95%CI: 0.31–0.65, *p* < 0.0001) (Figure 5). Median PFS were also significantly different between the 2 groups (2.0 vs 2.6 months respectively; HR: 0.59, 95%CI: 0.39–0.74, *p* = 0.0001).

**Figure 6:**
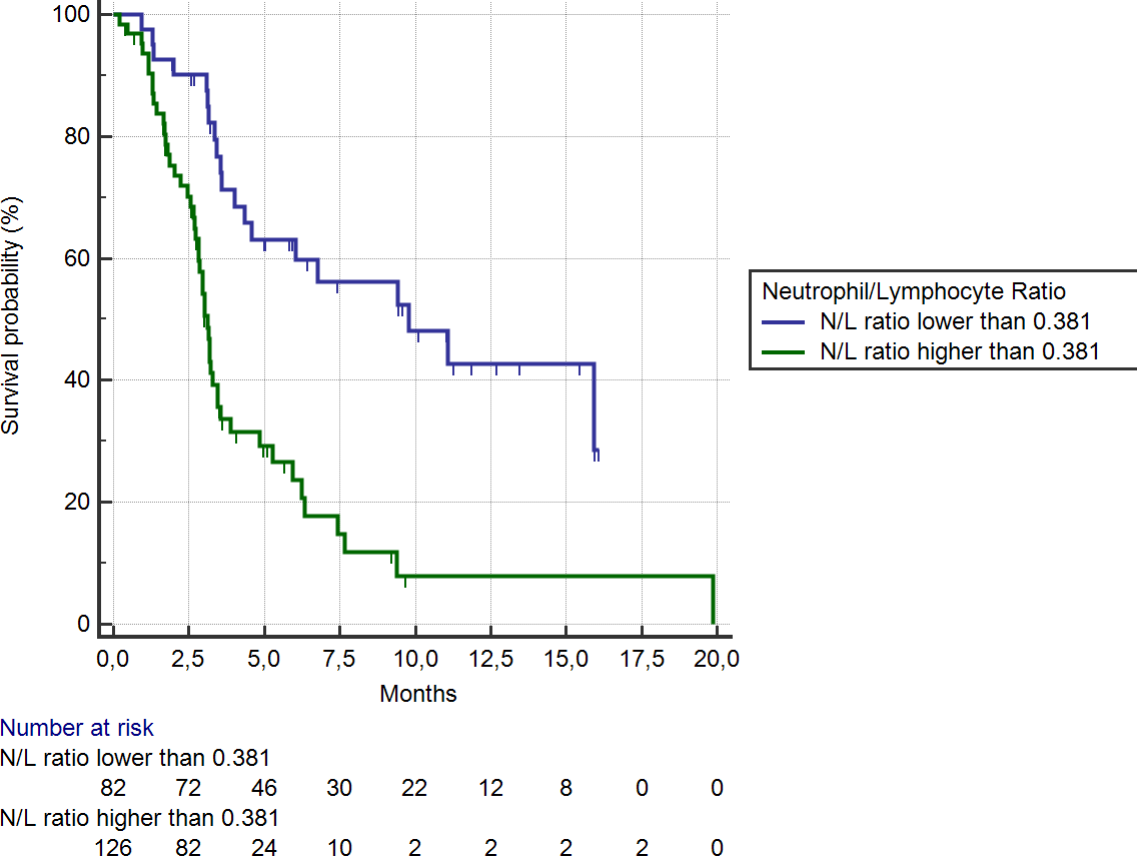
Kaplan-Meier curves for median overall survival (OS) in pre-treated metastatic colorectal cancer patients receiving regorafenib according to pre-treatment neutrophil/lymphocyte ratio < (**———**) or ≥ (**———**) than 0.38 (the cut-off value determined by ROC curve analysis) (9.8 months vs. 3.1 months; HR = 0.34, 95%CI: 0.22–0.45, *p* < 0.0001)

Eighty-two (39%) patients showed a NLR < 0.38 (the cut-off determined by ROC curve analysis). Median OS was 9.8 months vs. 3.1 months in patients with NLR < vs ≥ 0.38 respectively (HR: 0.34, 95%CI: 0.22–0.45, *p* < 0.0001) (Figure 6). Median PFS was also significantly different between the 2 groups (3.4 vs 2.1 months respectively, HR: 0.46, 95%CI:0.29–0.55, *p* < 0.0001).

All the other clinical variables analysed (age at diagnosis, gender, RAS mutation status, number of metastatic sites, previous adjuvant chemotherapy, number of previous systemic anticancer therapies) were not significantly related to clinical outcome (both for OS and PFS) (Table 1).

We also assessed whether dose reductions for any reason were related to a different outcome. About 25% patients experienced at least 1 dose reduction. In this group of patients no statistically significant differences were seen for overall survival (*p* = 0.066) or progression free survival (*p* = 0.44).

### Multivariate analysis and development of a risk score

At multivariate analysis all the factors that resulted significant at the univariate analysis (neutrophil count, lymphocyte count, platelet count and NLR) maintained their independent role as predictors of different OS, except for LDH levels. In particular, high platelet count (Exp(b):1.4963, 95%CI:1.0130–2.2103, *p* = 0.0439) and high NLR (Exp(b):1.6963, 95%CI:1.0757–2.6751, *p* = 0.0237) were related to worse OS, whereas a high lymphocyte level (Exp(b):0.4527, 95% CI:0.2801–0.7316, *p* = 0.0013) was related to better OS.

On the contrary the only 2 factors that maintained their roles as predictors of PFS were a high neutrophil level and a high NLR, with the latter showing the greatest impact on median PFS (Exp(b):1.7332, 95% CI:1.1752–2.5560, *p* = 0.0058).

By applying correction for multiple testing errors, all factors resulting independently related to overall survival maintained their statistical significance (lymphocyte count *p* = 0.0125, neutrophil count *p* = 0.0166, neutrophil/lymphocyte count *p* = 0.025, platelet count *p* = 0.05).

Among 52 (25%) patients who were negative for all risk factors, a significant correlation was found with improved OS and PFS if compared with the group of patients with at least 1 risk factor. In particular, median OS was respectively 15.9 vs 3.1 months (HR: 3.81, 95% CI: 2.32–4.82, *p* < 0.0001) whereas median PFS was 5.9 vs 2.1 months (HR: 2.62, 95% CI: 2.06–3.86, *p* < 0.0001). (Table 2).

**Table 2:** Median overall survival (mOS) for different groups of patients stratified according to the presence of the different independent prognostic factors as resulted from multivariate analysis (high platelets count, high neutrophil count, low lymphocyte count, high neutrophil/lymphocyte ratio); *p < 0.0001*

	Score			
	0	1	2	3
**Number of patients**	52	48	60	48
**mOS *(months)***	15.9	3.1	3.4	2.8

Legend

0: patients negative for all risk factors (patients with low platelets count, low neutrophil count, high lymphocyte count, low neutrophil/lymphocyte ratio)

1: patients with 1 risk factor among the independent prognostic factors above mentioned

2: patients with 2 risk factors among the independent prognostic factors above mentioned

3: patients with 3 risk factors among the independent prognostic factors above mentioned

On the basis of the results of the multivariate analysis, a prognostic score was developed.

**Figure 7:**
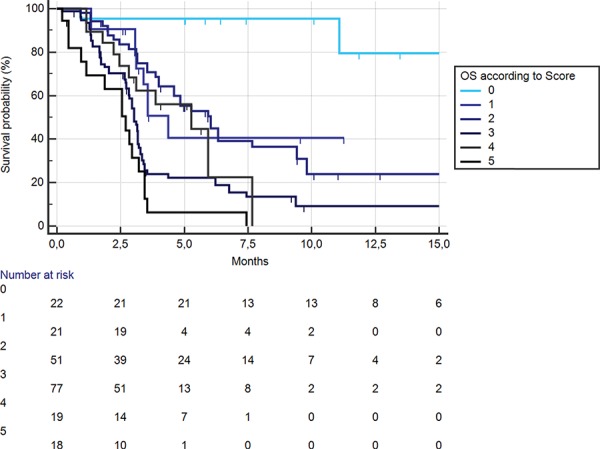
Kaplan-Meier curves for median overall survival (OS) in pre-treated metastatic colorectal cancer patients receiving regorafenib according to risk scoring: high PLT (+1), high neutrophil (+1), high neutrophil/lymphocyte (+1), low lymphocyte (+2)

In particular, for the presence of each a risk factor (high neutrophil/lymphocyte count, high platelet count, high neutrophil count) the patients’ risk score was raised by 1. Furthermore, the absence of high lymphocyte count was considered as another 2 point increase in the scoring, due to the greater role as protective factor of lymphocyte count. On this basis, patients’ scoring could range between 0 (extremely favourable) to 5 (extremely unfavourable). When overall survival was assessed on the basis of the scoring system, patients with extremely favourable prognosis had risk of death compared with patients with just 1 risk factor about 6 times lower (HR:0.1627, 95% CI:0.08454–0.3130, *p* < 0.0001). This difference was even greater when considering other risk groups (Figure 7). This relatively small group of patients (22/208, 11%) had a median overall survival that was not still reached at the time of data analysis.

## DISCUSSION

Despite regorafenib exhibited a small, but indisputable survival benefit in CRC patients not suitable for any further active treatment, the relevant toxicity profile along with the virtual absence of predictive factors suggested a careful evaluation of the benefit to risk ratio before widespread use in clinical practice.

The results of our analysis supports the idea that high pre-treatment NLR, high platelet and neutrophil count, and a low lymphocyte count are significantly and independently associated with worse clinical outcome in mCRC patients treated with regorafenib.

This is not surprising as it is generally accepted and demonstrated that the inflammatory process involving tumour microenvironment plays a crucial role in promoting proliferation, invasion and metastases of malignant cells [16, 32]. The infiltrating leucocytes, including neutrophils and lymphocytes, are critical factors for this biological process. Different proangiogenic factors such as the vascular endothelial growth factors originate from neutrophils and platelets-induced stimulation in the peripheral blood or in the tumour microenvironment and are eventually responsible for tumour progression [33]. Various clinical data highlighted the role of platelet levels in mCRC: a pre-planned sub-group analysis from the COIN trial, for example, suggested that patients with a high platelet count may not be optimal candidates for an intermittent strategy, thus remarking the poor prognostic profile of these patients [34].

Lymphocytes have been described as crucial components of the adaptive immune system and as the cellular basis of cancer immunosurveillance and immunoediting [35]. As a biological consequence, tumour infiltrating lymphocytes (TILs) have been frequently reported as indicator of an effective anti-tumour cellular immune response [36]. A low lymphocyte count may be responsible for an inadequate host-to-tumour immunologic reaction with a consequently decreased response against cancer, leading to poor clinical outcome.

The NLR has been already evaluated as prognostic factor in many tumour types as well as in CRC, usually related to an adverse outcome [17–23]. According to our data, NLR has been also indicated as a potentially relevant prognostic/predictive factor in CRC patients receiving an anti-angiogenic treatment such as bevacizumab [31].

In presence of stronger predictors of worse outcome, high LDH lost its independent role in influencing survival. In contrast to previous analyses in colorectal cancer patients receiving anti-angiogenic treatment in our experience high LDH serum levels indicated a trend toward a worse clinical outcome, rather than representing a predictive factor for response [11, 12]. We believe that the different setting analysed in our analysis might explain this apparent discrepancy. We can speculate that in early lines of treatment hypoxic factors might directly influence circulating isoforms of LDH such as LDH5 as predictors of efficacy to anti-VEGF therapy. On the contrary in latter lines of treatment, high levels of unselected LDH might be related to the heavy involvement of vital organs, such as the liver, regardless of the presence of an hypoxic drive.

Due to the independent prognostic role of all factors who have been included into the analysis, we hypothised that patients sub-groups with different clinical outcome during regorafenib treatment could be identified by taking into account all these factors combined. In particular, patients exhibiting a favourable profile (i.e. those without poor predictive indicators) seemed to benefit the most from the use of regorafenib.

The retrospective nature of this analysis and the lack of a proper control group (patients analysed for all the factors taken into account that received only BSC opposed to regorafenib treatment) prevents from drawing definitive conclusions on the prognostic or predictive value of the clinical factors analysed. It must be said that our analysis showed, in the group of patients who did not have any risk factor, not only an improved OS but also a significantly improved PFS. This latter observation may suggest a predictive more than a prognostic value of such factors.

Based on our findings, we can speculate that mCRC patients showing pre-treatment high neutrophil level, high platelet level, low lymphocytes level or high NLR may not be optimal candidates for regorafenib treatment. In these patients the risk of toxicity may not be in fact entirely balanced by the presumably poor clinical benefit deriving from treatment.

We believe that, after confirmation in further prospectively stratified and larger series, these clinical factors may play a relevant role in guiding treatment decision and prognostic stratification for patients receiving regorafenib. It would also be interesting to verify whether other factors (such as regorafenib starting dose or lack of anti-VEGF therapy in previous lines) might affect survival during regorafenib monotherapy. However this correlation was not possible in our paper as all patients included had already received bevacizumab and were started on regorafenib at the full dose of 160 mg/die).

Laboratory exams such as white blood cell count have a substantial advantage if compared with, although “fashionable”, biomolecular analyses: reproducibility and simplicity to obtain results without the need to perform more invasive tests. This could prove crucial in a setting where the identification of different risk-groups represents a key-challenge for the treatment of this disease, particularly now that different options are available and many more are hopefully to come.

## MATERIALS AND METHODS

### Patients selection

Histologically-proven mCRC patients who received regorafenib monotherapy after failure of previous chemotherapy regimens based on all standard drugs active in this setting (oxaliplatin, irinotecan, 5FU, bevacizumab and either cetuximab or panitumumab in case of RAS wild type tumour) were eligible for analysis.

Patients received regorafenib into the CORRECT and CONSIGN trials at our Institution (Ancona, Italy), at the Medical Oncology Department of the “Fondazione Poliambulanza” (Brescia, Italy), or at the Oncology Department of the “Azienda Ospedaliero-Universitaria Pisana” (Pisa, Italy). All patients had already received treatment with bevacizumab, oxaliplatin, irinotecan, 5FU/capecitabine and, in K-ras/RAS wild type patients, anti-EGFR monoclonal antibodies.

Treatment schedule was as follows: regorafenib 160 mg/die (day 1 to 21 every 28 days). Tumour response was evaluated every 8 weeks by clinicians’ assessment and according to the Response Evaluation Criteria in Solid Tumors (RECIST 1.1). LDH serum levels, neutrophil, lymphocyte and platelets count were collected within one month before the start of treatment. All patients should have started treatment at the dose of 160 mg/die, subsequent dose reductions were performed on the basis of tolerance to treatment as assessed by treating clinicians.

### Laboratory exams specifics

All laboratory exams where determined according to IFCC (International Federation of Clinical Chemistry and Laboratory Medicine) method. The assay has been conducted in Institution Laboratories certified for Quality control according to the present rules in Europe.

For study purposes, LDH levels for each patient was calculated as the ratio between LDH serum levels at the beginning of the treatment with regorafenib and the upper-normal-limit (UNL) for the laboratory where the test was performed.

Neutrophil level was determined as the ratio between neutrophil level at the treatment start and the upper-normal-limit (UNL) for the laboratory where the test was performed. Lymphocyte level was determined as the ratio between lymphocyte level at the treatment start and the lower-limit-normal (LLN) for the laboratory where the test was performed.

Platelets level was determined as the ratio between platelets level at the treatment start and the upper-normal-limit (UNL) for the laboratory where the test was performed.

By performing the ratio between the point value of the single test by the LLN and UNL, we standardized the values of the determinations between the 3 laboratories where tests were performed.

Cut-off values for LDH, neutrophil, lymphocyte, NLR and platelet levels were determined by receiver operating characteristics (ROC) curve analysis in the whole patients population, assuming PFS (>2 months) as a classification variable.

### Statistical analysis

Statistical analysis was performed with the MedCalc package (MedCalc^®^ v9.4.2.0).

The association between categorical variables was assessed by chi-square test.

Survival distribution was estimated by the Kaplan-Meier method. Significant differences in probability of relapsing between the strata were evaluated by log-rank test. Cox multiple regression analysis was used to assess the role of variables resulted significant at univariate analysis (multivariate analysis).

Other tested variables included gender (male vs. female), age (<65 yrs vs. ≥ 65 yrs), RAS mutational status (wild type vs. mutated), metastatic sites (< 2 vs. ≥ 2), previous adjuvant chemotherapy, previous systemic anticancer therapy (≤ 2 vs. 3 vs. ≥ 4).

A significant level of 0.05 was chosen to assess the statistical significance.

For statistical analysis, OS and PFS were defined respectively as the interval between the start of treatment to death or last follow-up visit and as the interval between the start of treatment to clinical progression or death or last follow up visit if not progressed.

Correction for multiple testing errors has been performed by the Holm-Sidak method.

## References

[1] Grothey A, Van Cutsem E, Sobrero A, Siena S, Falcone A, Ychou M, Humblet Y, Bouché O, Mineur L, Barone C, Adenis A, Tabernero J, Yoshino T (2013). Regorafenib monotherapy for previously treated metastatic colorectal cancer (CORRECT): an international, multicentre, randomised, placebo-controlled, phase 3 trial. Lancet.

[2] Maxwell PH, Pugh CW, Ratcliffe PJ (2001). Activation of the HIF pathway in cancer. Curr Opin Genet Dev.

[3] Koukourakis MI, Giatromanolaki A, Sivridis E (2003). Lactate dehydrogenase isoenzymes 1 and 5: differential expression by neoplastic and stromal cells in non-small cell lung cancer and other epithelial malignant tumors. Tumour Biol.

[4] Harris AL (2002). Hypoxia—a key regulatory factor in tumour growth. Nat Rev Cancer.

[5] Azuma M, Shi M, Danenberg KD, Gardner H, Barrett C, Jacques CJ, Sherod A, Iqbal S, El-Khoueiry A, Yang D, Zhang W, Danenberg PV, Lenz HJ (2007). Serum lactate dehydrogenase levels and glycolysis significantly correlate with tumor VEGFA and VEGFR expression in metastatic CRC patients. Pharmacogenomics.

[6] Tas F, Aykan F, Alici S, Kaytan E, Aydiner A, Topuz E (2001). Prognostic factors in pancreatic carcinoma: serum LDH levels predict survival in metastatic disease. Am J Clin Oncol.

[7] Tas F, Aydiner A, Demir C, Topuz E (2001). Lactate dehydrogenase levels at presentation predict outcome of patients with limited stage small-cell lung cancer. Am J Clin Oncol.

[8] Koukourakis MI, Giatromanolaki A, Simopoulos C, Polychronidis A, Sivridis E (2005). Lactate dehydrogenase 5 (LDH5) relates to up-regulated hypoxia inducible factor pathway and metastasis in colorectal cancer. Clin Exp Metastasis.

[9] Wu XZ, Ma F, Wang XL (2010). Serological diagnostic factors for liver metastasis in patients with colorectal cancer. World J Gastroenterol.

[10] Hecht JR, Trarbach T, Hainsworth JD, Major P, Jäger E, Wolff RA, Lloyd-Salvant K, Bodoky G, Pendergrass K, Berg W, Chen BL, Jalava T, Meinhardt G, Laurent D, Lebwohl D, Kerr D (2011). Randomized, Placebo-Controlled, Phase III Study of First-Line Oxaliplatin-Based Chemotherapy Plus PTK787/ZK222584, an Oral Vascular Endothelial Growth Factor Receptor Inhibitor, in Patients With Metastatic Colorectal Adenocarcinoma. J Clin Oncol.

[11] Van Cutsem E, Bajetta E, Valle J, Köhne CH, Hecht JR, Moore M, Germond C, Berg W, Chen BL, Jalava T, Lebwohl D, Meinhardt G, Laurent D, Lin E (2011). Randomized, placebo-controlled, phase III study of Oxaliplatin, Fluorouracil and Leucovorin with or without PTK787/ZK222584 in patients with previously treated metastatic colorectal adenocarcinoma. J Clin Oncol.

[12] Koukourakis MI, Giatromanolaki A, Sivridis E, Gatter KC, Trarbach T, Folprecht G, Shi MM, Lebwohl D, Jalava T, Laurent D, Meinhardt G, Harris AL (2011). Prognostic and predictive role of lactate dehydrogenase 5 expression in colorectal cancer patients treated with PTK787/ZK 222584 (vatalanib) antiangiogenic therapy. Clin Cancer Res.

[13] Scartozzi M, Giampieri R, Maccaroni E, Del Prete M, Faloppi L, Bianconi M, Galizia E, Loretelli C, Belvederesi L, Bittoni A, Cascinu S (2012). Pre-treatment lactate dehydrogenase levels as predictor of efficacy of first-line bevacizumab-based therapy in metastatic colorectal cancer patients. Br J Cancer.

[14] Colotta F, Allavena P, Sica A, Garlanda C, Mantovani A (2009). Cancer-related inflammation, the seventh hallmark of cancer: links to genetic instability. Carcinogenesis.

[15] Hanahan D, Weinberg RA (2011). Hallmarks of cancer: the next generation. Cell.

[16] Mantovani A, Allavena P, Sica A, Balkwill F (2008). Cancer-related inflammation. Nature.

[17] Proctor MJ, McMillan DC, Morrison DS, Fletcher CD, Horgan PG, Clarke SJ (2012). A derived neutrophil to lymphocyte ratio predicts survival in patients with cancer. Br J Cancer.

[18] Walsh SR, Cook EJ, Goulder F, Justin TA, Keeling NJ (2005). Neutrophil-lymphocyte ratio as a prognostic factor in colorectal cancer. J Surg Oncol.

[19] Paramanathan A, Saxena A, Morris DL (2014). A systematic review and meta-analysis on the impact of pre-operative neutrophil lymphocyte ratio on long term outcomes after curative intent resection of solid tumours. Surg Oncol.

[20] Ishizuka M, Nagata H, Takagi K, Iwasaki Y, Kubota K (2013). Combination of platelet count and neutrophil to lymphocite ratio is a useful predictor of postoperative survival in patients with colorectal cancer. Br J Cancer.

[21] Absenger G, Szkandera J, Stotz M, Postlmayr U, Pichler M, Ress AL, Schaberl-Moser R, Loibner H, Samonigg H, Gerger A (2013). Preoperative neutrophil-to-lymphocyte ratio predicts clinical outcome in patients with stage II and III colon cancer. Anticancer Res.

[22] Absenger G, Szkandera J, Pichler M, Stotz M, Arminger F, Weissmueller M, Schaberl-Moser R, Samonigg H, Stojakovic T, Gerger A (2013). A derived neutrophil to lymphocyte ratio predicts clinical outcome in stage II and III colon cancer patients. Br J Cancer.

[23] Li MX, Liu XM, Zhang XF, Zhang JF, Wang WL, Zhu Y, Dong J, Cheng JW, Liu ZW, Ma L, Lv Y (2014). Prognostic role of neutophil to lymphocyte ratio in colorectal cancer: a systematic review and meta-analysis. Int J Cancer.

[24] Milasiene V, Stratilatovas E, Norkiene V, Jonusauskaite R (2005). Lymphocyte subsets in peripheral blood as prognostic factors in colorectal cancer. J Buon.

[25] Fogar P, Sperti C, Basso D, Sanzari MC, Greco E, Davoli C, Navaglia F, Zambon CF, Pasquali C, Venza E, Pedrazzoli S, Plebani M (2006). Decreased total lymphocyte counts in pancreatic cancer: an index of adverse outcome. Pancreas.

[26] Saroha S, Uzzo RG, Plimack ER, Ruth K, Al-Saleem T (2013). Lymphopenia is an independent predictor of inferior outcome in clear cell renal carcinoma. J Urol.

[27] Lissoni P, Fumagalli L, Brivio F, Rovelli F, Messina G, Di Fede G, Colciago M, Brera G (2006). Cancer chemotherapy-induced lymphocytosis: a revolutionary discovery in the medical oncology. J Biol Regul Homeost Agents.

[28] Kishi Y, Kopetz S, Chun YS, Palavecino M, Abdalla EK, Vauthey JN (2009). Blood neutrophil to lymphocyte ratio predicts survival in patients with colorectal liver metastases treated with systemic chemotherapy. Ann Surg Oncol.

[29] Chua W, Charles KA, Baracos VE, Clarke SJ (2011). Neutrophil/lymphocyte ratio predicts chemotherapy outcomes in patients with advanced colorectal cancer. Br J Cancer.

[30] Guillem-Llobat P, Dovizio M, Alberti S, Bruno A, Patrignani P (2014). Platelets, cyclooxygenases, and colon cancer. Semin Oncol.

[31] Clarke S, Burge M, Cordwell C, Gibbs P, Reece W, Tebbutt N (2013). An australian translational study to evaluate the prognostic role of inflammatory markers in patients with metastatic ColorEctal caNcer Treated with bevacizumab (Avastin)[ASCENT]. BMC Cancer.

[32] Proctor MJ, Morrison DS, Talwar D, Balmer SM, Fletcher CD, O'Reilly DS, Foulis AK, Horgan PG, McMillan DC (2011). A comparison of inflammation-based prognostic scores in patients with cancer. A Glasgow Inflammation Outcome Study. Eur J Cancer.

[33] Kusumanto YH, Dam WA, Hospers GA, Meijer C, Mulder NH (2003). Platelets and granulocytes, in particular the neutrophils, form important compartments for circulating vascular endothelial growth factor. Angiogenesis.

[34] Adams RA, Meade AM, Seymour MT, Wilson RH, Madi A, Fisher D, Kenny SL, Kay E, Hodgkinson E, Pope M, Rogers P, Wasan H, Falk S (2011). Intermittent versus continuous oxaliplatin and fluoropyrimidine combination chemotherapy for first-line treatment of advanced colorectal cancer: results of the randomised phase 3 MRC COIN trial. Lancet Oncol.

[35] Dunn GP, Old LJ, Schreiber RD (2004). The immunobiology of cancer immunosurveillance and immunoediting. Immunity.

[36] Rabinowich H, Cohen R, Bruderman I, Steiner Z, Klajman A (1987). Functional analysis of mononuclear cells infiltrating into tumours: lysis of autologous human tumour cells by cultured infiltrating lymphocytes. Cancer Res.

